# Activation of MAPK/ERK signaling by *Burkholderia pseudomallei* cycle inhibiting factor (Cif)

**DOI:** 10.1371/journal.pone.0171464

**Published:** 2017-02-06

**Authors:** Mei Ying Ng, Mei Wang, Patrick J. Casey, Yunn-Hwen Gan, Thilo Hagen

**Affiliations:** 1 Department of Biochemistry, Yong Loo Lin School of Medicine, National University of Singapore, Singapore; 2 Program in Cancer and Stem Cell Biology, Duke-NUS Graduate Medical School, Singapore, Singapore; Medical College of Wisconsin, UNITED STATES

## Abstract

Cycle inhibiting factors (Cifs) are virulence proteins secreted by the type III secretion system of some Gram-negative pathogenic bacteria including *Burkholderia pseudomallei*. Cif is known to function to deamidate Nedd8, leading to inhibition of Cullin E3 ubiquitin ligases (CRL) and consequently induction of cell cycle arrest. Here we show that Cif can function as a potent activator of MAPK/ERK signaling without significant activation of other signaling pathways downstream of receptor tyrosine kinases. Importantly, we found that the ability of Cif to activate ERK is dependent on its deamidase activity, but independent of Cullin E3 ligase inhibition. This suggests that apart from Nedd8, other cellular targets of Cif-dependent deamidation exist. We provide evidence that the mechanism involved in Cif-mediated ERK activation is dependent on recruitment of the Grb2-SOS1 complex to the plasma membrane. Further investigation revealed that Cif appears to modify the phosphorylation status of SOS1 in a region containing the CDC25-H and proline-rich domains. It is known that prolonged Cullin E3 ligase inhibition leads to cellular apoptosis. Therefore, we hypothesize that ERK activation is an important mechanism to counter the pro-apoptotic effects of Cif. Indeed, we show that Cif dependent ERK activation promotes phosphorylation of the proapoptotic protein Bim, thereby potentially conferring a pro-survival signal. In summary, we identified a novel deamidation-dependent mechanism of action of the *B*. *pseudomallei* virulence factor Cif/CHBP to activate MAPK/ERK signaling. Our study demonstrates that bacterial proteins such as Cif can serve as useful molecular tools to uncover novel aspects of mammalian signaling pathways.

## Introduction

Cycle inhibiting factors (Cifs) are a group of bacterial virulence proteins that are secreted by the Type III Secretion System of some Gram-negative bacterial pathogens including *Burkholderia pseudomallei* and enteropathogenic and enterohemorrhagic *Escherichia coli* [1,2]. Upon injection into host cells, Cif is known to inhibit host cell cycle progression at both G_1_/S and G_2_/M phase transitions. It has been shown that the cell cycle arrest is induced as a result of stabilization of cyclin-dependent kinase inhibitors p21^waf1/cip1^ and p27^kip1^ (hereafter referred to as p21 and p27) [3–6]. Cellular concentrations of the cell cycle inhibitors p21 and p27 are normally tightly regulated via ubiquitination by cullin RING E3 ubiquitin ligases (CRLs). Ubiquitinated p21 and p27 are then targeted to the 26S proteasome for degradation [7–9]. Cif has been shown to inhibit CRL function, leading to the accumulation of p21, p27 and numerous other CRL substrates.

CRLs constitute the largest family of E3 ubiquitin ligases, primarily due to their modular structure. There are six different Cullin homologous proteins, with each forming a scaffold onto which different E3 ligase complexes assemble [10,11]. CRLs bind the RING subunit at the carboxy-terminus, which functions to recruit the ubiquitin-charged E2 ubiquitin conjugating enzyme. At the amino-terminus, CRLs bind different substrate receptors, which are responsible for recruiting specific cellular substrate proteins. As a result of the assembly, the substrate is brought into close proximity to the ubiquitin-charged E2 enzyme, thus facilitating the transfer of ubiquitin from the E2 enzyme onto the substrate. However, CRL-dependent ubiquitination of substrate proteins requires modification of CRL by the 76-amino acid ubiquitin-like protein Nedd8. Nedd8 conjugation to a conserved lysine residue in the carboxy-terminus of CRLs induces a conformational change in CRL that is required to activate its ubiquitination activity.

Recent studies have revealed that Cif functions as a deamidase that targets Nedd8 on Gln40, thus converting it into a glutamate [4]. Mechanistically, we and others have shown that Nedd8 deamidation does not preclude the conjugation of Nedd8 onto the Cullin protein, but prevents the Nedd8 induced conformational change of the CRL complex [12,13]. Consequently, the Cif-mediated enzymatic modification in Nedd8 results in a marked inhibition of CRL activity, thus preventing ubiquitination of substrate proteins by CRLs and leading to stabilization of p21, p27 and other CRL substrates. Hence, the cell cycle inhibitory effect of Cif is attributed to Cif-dependent inhibition of CRL activity involving Nedd8 deamidation.

Here we show that in addition to Nedd8 deamidation, Cif also has CRL independent effects. Thus, we found that Cif induces a potent and selective activation of the pro-survival mitogen-activated protein kinase/extracellular signal-regulated kinase (MAPK/ERK) pathway. We sought to characterize the mechanism through which Cif activates ERK. We show that Cif-mediated ERK activation is dependent on its deamidase activity but independent of CRL inhibition, suggesting that the MAPK/ERK pathway is a novel target of Cif that is different from CRL. Our results show that Cif targets the SOS1-Grb2 complex in the MAPK/ERK pathway and modifies the phosphorylation status of the Ras guanine nucleotide exchange factor, SOS1, specifically in the region of SOS1 containing the CDC25-homology and proline-rich domains. We suggest that Cif-mediated ERK activation functions to counter the proapoptotic effects of CRL inhibition.

## Materials and methods

### Cell culture and transfection

HEK293T cells were cultured in Dulbecco’s modified eagle medium (DMEM) (Invitrogen) supplemented with 10% (vol/vol) heat-inactivated fetal bovine serum (Hyclone), 2 mM L-glutamine (Invitrogen), 100 U/ml penicillin and 100 μg/ml streptomycin (Invitrogen) in a humidified 37°C, 5% CO_2_ tissue culture incubator. Transient transfections were performed using Genejuice transfection reagent (Novagen) in accordance with the manufacturer’s directions for sub-confluent cells. MLN4924 was a gift from Millennium Pharmaceuticals, Inc.

### Plasmid constructs

The FLAG- or V5-*B*. *pseudomallei* Cif plasmids bearing an N-terminal 2X FLAG or V5 epitope tag sequence used were previously described [12]. *B*. *pseudomallei* Cif C156S catalytic mutant (V5-Cif C156S) was generated via site-directed mutagenesis using forward primer 5’-GAT GAC GCC CGT GTC CGG ACT TTC GGC CA-3’ and reverse primer 5’-TGG CCG AAA GTC CGG ACA CGG GCG TCA TC-3’, and cloned into pcDNA3.1 with 5’ KpnI and 3’ XbaI and verified by DNA sequencing. FLAG-Bim_EL_ plasmid was a kind gift from Dr. Ong Sin Tiong (Duke NUS Medical School). pBabe-Puro-MEK-DD and pBabe-Puro-B-RAF-V600E were gifts from Dr. William Hahn (Dana Farber Cancer Institute) (Addgene plasmid # 15268 and # 15269 respectively) [14]. The FLAG-WT HRas and FLAG-G12V HRas plasmids were generated by amplification from mEGFP-HRas and mEGFP-HRas G12V (gifts from Dr. Karel Svoboda (Janelia Research Campus), Addgene plasmid # 18662 and # 18666, respectively) [15] and cloned into pcDNA3.1 with 5’ KpnI and 3’ XbaI as described above. FLAG-S17N HRas, dn SH2, SH3N (N-terminal SH3 domain) and SH3C (C-terminal SH3 domain) Grb2-HA plasmids were generated via site-directed mutagenesis and verified by DNA sequencing. FLAG-mSOS1 plasmid was a kind gift from Dr. Low Boon Chuan (National University of Singapore). FLAG-mSOS1 truncation mutants were generated by PCR through sequential C-terminal truncations.

### Immunoblotting

Cells were washed with ice-cold Phosphate-Buffered Saline (PBS) and then lysed in Triton X-100-containing lysis buffer. The composition of the lysis buffer was as follows: 25 mM Tris-HCl (pH 7.5), 100 mM NaCl, 2.5 mM EDTA, 2.5 mM EGTA, 20 mM NaF, 1 mM Na_3_VO_4_, 20 mM Sodium β-Glycerophosphate, 10 mM Sodium Pyrophosphate, 0.5% Triton X-100, Roche protease inhibitor cocktail and 0.1% β-Mercaptoethanol. Lysates were precleared by centrifugation before use for Western blotting. Equal amounts of protein were loaded for Western blot analysis. All the following antibodies used were obtained from Cell Signaling Technology: anti-phospho-p38 (Thr180/Tyr182), anti-p38, anti-phospho-SAPK/JNK (Thr183/Tyr185), anti-SAPK/JNK, anti-phospho-ERK1/2 (Thr202/Tyr204), anti-ERK1/2, anti-Mcl-1, anti-phospho-Bad (Ser112), anti-Bad, anti-phospho-MEK1/2, anti-MEK1/2, anti-Grb2, anti-phospho-Akt (Thr308), anti-Akt, anti-phospho-STAT3 (Tyr705), anti-STAT3, anti-calnexin, anti-phospho-p70 S6K1 (Thr389), anti-p70S6K1, except for anti-p27 (BD Biosciences), anti-HIF-1α (BD Transduction Laoratories), anti-GAPDH (US Biological), anti-Glut1 (Abcam), anti-α-tubulin (Molecular Probes), anti-β-actin (Sigma), anti-FLAG M2 (Sigma), anti-V5 (Serotec) and anti-HA (Roche).

### Immunoprecipitation

20 μl of anti-FLAG M2 agarose beads (Sigma) were used for immunoprecipitation. 500 μl of precleared lysate from cells transfected with FLAG-mSOS1 + empty vector or FLAG-mSOS1 + V5-Cif in 60-mm tissue culture dishes was added to the agarose beads. Untransfected cell lysate was added to control beads. The samples were tumbled for 1 hour at 4°C, and the beads were then washed four times in ice-cold NP40 lysis buffer (containing 50 mM NaCl, 0.5% NP-40, 5% glycerol, 0.5 mM EDTA, 50 mM Tris, pH 7.5) and once in ice-cold buffer containing 50 mM Tris (pH 7.5). The immunoprecipitated mSOS1 proteins were then eluted and denatured in 2X Laemmli sample buffer (Biorad) containing 5% β-Mercaptoethanol and subjected to SDS-PAGE and Western blotting.

## Results

### Cif increases ERK MAPK phosphorylation in a manner dependent on its deamidase activity but independent of CRL inhibition

**Fig 1:**
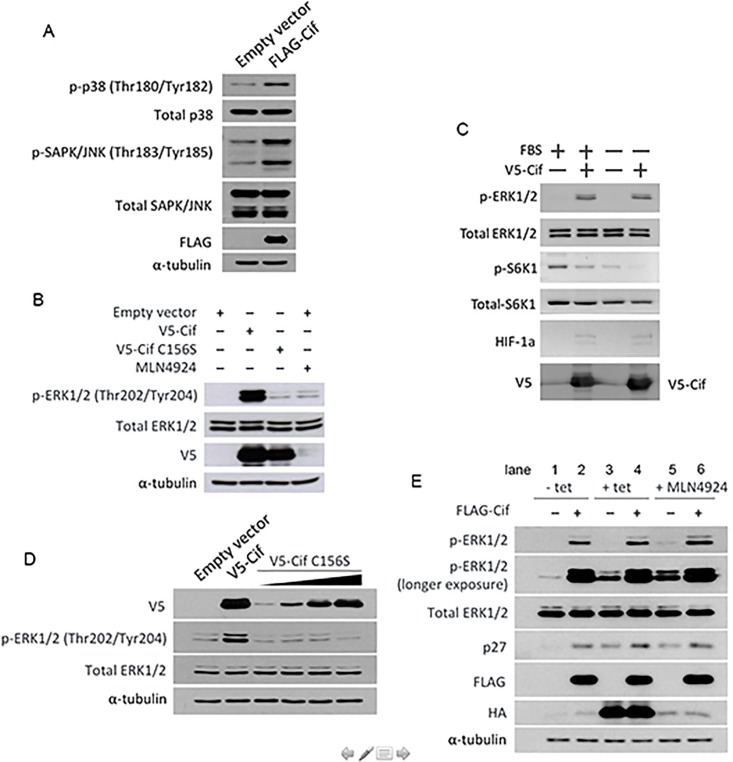
Cif increases ERK MAPK phosphorylation in a manner dependent on its deamidase activity but independent of CRL inhibition. (A,B) HEK293T cells were transfected with the indicated expression plasmids for two days, (B) and treated with 1 μM MLN4924 for the last 24 hours. Pre-cleared cell lysates were then subjected to Western blotting analysis with the indicated antibodies. (C) HEK293T cells were transfected for two days with V5-Cif, as indicated. Cells were then serum-starved for 3 hours, followed by cell lysis and Western blot analysis with the indicated antibodies. The phospho-p70 S6K1 Western blot served as a positive control for the effect of serum starvation. As expected, growth factor withdrawal resulted in reduced p70 S6K1 phosphorylation. It was also noted that Cif expression had an inhibitory effect on p70 S6K1 phosphorylation both in the presence and absence of serum. (D) Cells were transfected with the indicated expression plasmids. For V5-Cif C156S expression, increasing amounts were transfected whereby the lowest amount equaled that of the wild type V5-Cif expression plasmid. Cell lysates were analysed by Western blotting using the indicated antibodies. (E) Tetracycline-inducible dominant negative Ubc12 HEK293 cells were transfected with FLAG-Cif expression plasmid for two days. Cells were then either treated with 1 μM tetracycline to induce dnUbc12 expression for the last 24 hours or treated with 1 μM MLN4924 for the last 24 hours. Cell lysates were then analysed by Western blotting using the indicated antibodies.

In our efforts to discover mechanisms of action of Cif that are independent of CRL inhibition, we observed that Cif has a potent activating effect on the ERK pathway (see Fig 1B and Ng *et al*., manuscript in preparation). Therefore, we sought to determine the exact molecular target and mechanism of action of Cif in regulating ERK activity. We first determined if Cif exhibits a preference for activating the ERK pathway compared to the p38 or stress-activated protein kinase/c-Jun amino terminal kinase (SAPK/JNK) MAPK pathways. Ectopic expression of Cif using a FLAG- or V5-epitope tagged Cif plasmid in HEK293T cells resulted in a marked induction of ERK1/2 phosphorylation both (Fig 1A–1C). In contrast, Cif caused only a slight increase in p38 and SAPK/JNK phosphorylation (Fig 1A and 1B). Activation of ERK1/2 by Cif was observed both in the presence and absence of serum (Fig 1C) and was observed concomitantly with accumulation of CRL substrates HIF-1α (Fig 1C) and p27 (see Fig 1E). Thus, it can be concluded that Cif selectively and potently activates the MAPK/ERK pathway.

Cif has recently been reported to function as a deamidase, and its deamidase activity is dependent on its catalytic triad [4]. Hence, we next sought to determine if the effect of Cif on ERK phosphorylation is dependent on its deamidase activity. To test this, we expressed a mutant of Cif in which the catalytic cysteine 156 residue in the triad is mutated to serine (C156S), in cells. As shown in Fig 1B, the catalytic inactive Cif mutant did not increase ERK1/2 phosphorylation, suggesting that ERK activation by Cif is dependent on its deamidase activity. It was noted that the expression level of the Cif mutant is not identical to that of wild type Cif. Hence, to confirm that the lack of effect of the Cif mutant is not due to the lower expression level, we performed a titration of the Cif mutant and measured the level of ERK activation. We found that a dose-dependent increase in the expression of the Cif mutant did not result in a corresponding increase in ERK activation (Fig 1D). This further supports the finding that the Cif mutant is impaired in activating ERK, and that the effect of Cif on ERK activation is dependent on its deamidase activity.

To date, the only known mechanism of action of Cif is to inhibit CRL activity via deamidation of its substrate Nedd8 [4]. Therefore, we investigated whether the effect of Cif on ERK activation is a consequence of CRL inhibition. To test this, we used the CRL inhibitor MLN4924. MLN4924 blocks the activation of Nedd8 by the Nedd8 E1-activating enzyme [16]. This prevents the transfer of Nedd8 onto CRLs and blocks the conformational change that is required to activate CRLs. As shown in Fig 1B, when cells were treated with MLN4924, no increase in ERK activation was observed. This suggests that ERK activation is unlikely to be a consequence of CRL inhibition. To confirm this result, we used an alternative approach to inhibit CRL activity by employing a cell line that expresses a dominant negative mutant of the Nedd8 E2-conjugating enzyme Ubc12 (dnUbc12). In this cell line, dnUbc12 expression is under control of a tetracycline-inducible promoter [17]. Upon addition of tetracycline, dnUbc12 is induced and sequesters cellular Nedd8, thereby preventing endogenous Ubc12 from conjugating Nedd8 onto CRLs. As expected, expression of dnUbc12 resulted in the accumulation of the CRL substrate p27 (Fig 1E). As shown in Fig 1E, expression of Cif resulted in a similar increase in ERK1/2 phosphorylation in the absence and presence of tetracycline (lane 2 and 4). Thus, Cif causes marked ERK activation even when CRL activity is inhibited. Similarly, when Cif was expressed in the presence of the CRL inhibitor MLN4924, a further increase in ERK activity was observed (compare lane 6 to lane 5). Taken together, these results suggest that the effect of Cif on ERK activity is independent of CRL inhibition, but dependent on Cif deamidase activity. Therefore, we hypothesized that Cif activates the MAPK/ERK pathway via a target that is different from CRL.

### ERK activation confers a pro-survival signal through phosphorylation of the pro-apoptotic protein Bim

It has been postulated that the inhibition of cell cycle progression as a result of Cif-dependent CRL inhibition slows down the turnover rate of epithelial cells [18], thus promoting bacterial colonization of the lung epithelium. However, it is well known that prolonged CRL inhibition results in the accumulation of key cell cycle mediators and ultimately induction of cellular apoptosis. The induction of apoptosis would self-limit *B*. *pseudomallei* infection. This raises the question of how the bacterium simultaneously inhibits host cell proliferation and prevents cell apoptosis. Therefore, we hypothesized that the physiological role of Cif in activating the pro-survival MAPK/ERK pathway is to counter the proapoptotic effects of CRL inhibition.

**Fig 2:**
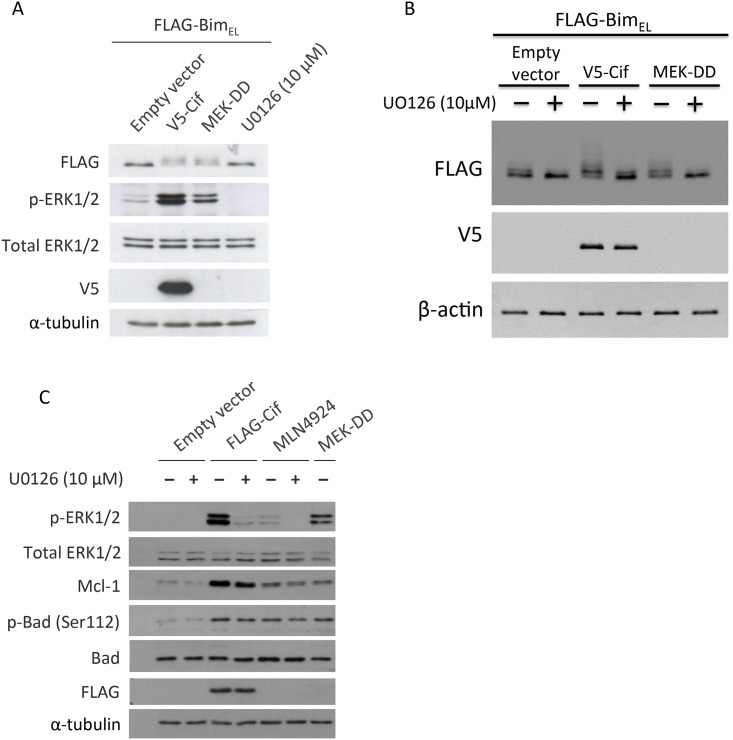
Cif regulates the expression or activity of apoptosis related proteins. (A) Cells were co-transfected with FLAG-Bim_EL_ and V5-Cif, MEK-DD or empty vector, or treated with 10 μM U0126 for the last 6 hours. Cell lysates were then subjected to Western blotting using the indicated antibodies. (B) Cells were co-transfected with FLAG-Bim_EL_ and V5-Cif, MEK-DD or empty vector and treated with 10 μM U0126 for the last 6 hours, as indicated, followed by Western blotting using the indicated antibodies. (C) Cells were transfected with FLAG-Cif, MEK-DD or empty vector and treated with 10 μM U0126 for the last 6 hours and with 1 μM MLN4924 for the last 24 hours, as indicated, followed by Western blotting using the indicated antibodies.

ERK is known to inhibit cellular apoptosis by regulating the phosphorylation status of the BH3-only protein Bim [19]. Therefore, we examined whether Cif functions to modulate Bim phosphorylation. There are three major isoforms of Bim that are generated as a result of alternative splicing: Bim short (Bim_s_), Bim long (Bim_L_), and Bim extra long (Bim_EL_) [19]. Bim_EL_ has been reported to be the most abundant isoform. This isoform has also been shown to contain an ERK1/2 docking domain and ERK1/2 phosphorylation sites. Phosphorylation of Bim_EL_ by ERK1/2 targets it to the proteasome for degradation. Therefore, to test whether Cif counters the effect of the proapoptotic protein Bim, we expressed FLAG-tagged Bim_EL_ in the presence or absence of Cif and measured the phosphorylation of Bim_EL_ by western blot. We also used a known activator of ERK MAPK, active MEK (MEK-DD), as a positive control, as well as the MEK1/2 inhibitor U0126 as a negative control. As shown in Fig 2A, a band shift in Bim_EL_ was detected when FLAG-Bim_EL_ was co-expressed with Cif, compared to the empty vector control in the first lane. A similar band shift was also observed when FLAG-Bim_EL_ was co-expressed with MEK-DD. Cif- and MEK-DD-induced Bim_EL_ phosphorylation was not observed when ERK1/2 activation by Cif and MEK-DD was prevented through treatment with U0126 (Fig 2B). This indicates that Cif-induced Bim_EL_ phosphorylation is dependent on ERK1/2. Of note, we also observed that Cif expression increased the protein levels of anti-apoptotic Mcl-1 and promoted inhibitory phosphorylation of the pro-apoptotic Bad protein at Serine 112 (Fig 2C). However, these effects of Cif were not inhibited by U0126 and were hence ERK1/2 independent. Moreover, inhibition of CRL activity with MLN4924 did not mimic the effects of Cif on Mcl-1 and Bad. Hence, the Cif induced increases in Mcl-1 protein expression and Bad Serine 112 phosphorylation are likely not due to CRL inhibition. Thus, when taken together our results suggest that Cif can exert anti-apoptotic effects through ERK1/2 dependent and independent mechanisms. This may play a role to counter the pro-apoptotic effects of Cif dependent CRL inhibition.

### Activation of the MAPK/ERK pathway by Cif is independent of MAPK phosphatases

**Fig 3:**
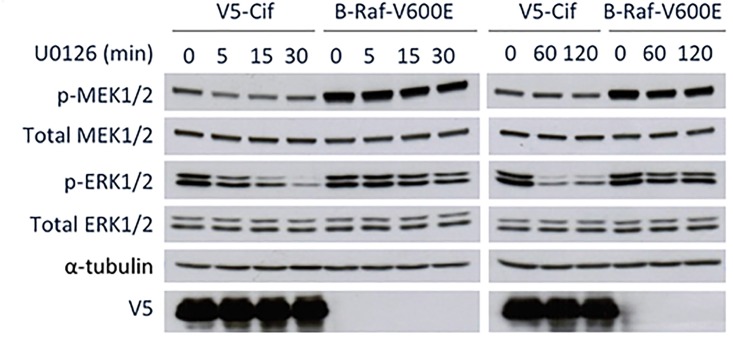
Cif activates MAPK/ERK signaling in a manner independent of MAPK phosphatases. Cells were transfected with V5-Cif or B-Raf-V600E and treated with 10 μM U0126 for the last 5 to 120 minutes, followed by Western blotting of the cell lysates using the indicated antibodies.

Cif could potentially induce ERK1/2 phosphorylation via two mechanisms—by inhibiting MAPK phosphatases or by activating ERK or upstream MAP kinases. To determine if Cif induces ERK activation by inhibiting MAPK phosphatases, we performed a chase experiment whereby we followed the rate of ERK dephosphorylation in the presence of the MEK inhibitor U0126. U0126 blocks the activity of phosphorylated MEK, thus rendering MEK unable to phosphorylate its downstream substrate ERK1/2. In the experiment in Fig 3, ERK1/2 phosphorylation was induced by transfecting cells with either Cif or constitutively active B-Raf-V600E. At time zero, cells were treated with U0126 to prevent further ERK1/2 phosphorylation. As shown in Fig 3, in cells expressing B-Raf-V600E, treatment with U0126 resulted in a decrease in ERK1/2 phosphorylation over time. In cells transfected with Cif, ERK1/2 phosphorylation decreased at a significantly faster rate. This result shows that it is unlikely that Cif functions by inhibiting MAPK phosphatases to prevent ERK dephosphorylation.

### Cif targets the MAPK/ERK pathway upstream of Ras and downstream of RTKs

To elucidate the target of Cif in the MAPK/ERK pathway, we took a systematic bottom-up approach. Thus, we examined if Cif directly activates a candidate kinase or a kinase that is upstream in the pathway by using a panel of kinase inhibitors. We found that when cells were treated with the MEK inhibitor U0126, Cif-dependent ERK activation was completely prevented (Fig 2C). U0126 blocks the ability of active MEK to phosphorylate ERK1/2. Therefore, the lack of effect of Cif on ERK phosphorylation in the presence of U0126 suggests that Cif does not directly activate ERK but functions upstream of the ERK kinase.

**Fig 4:**
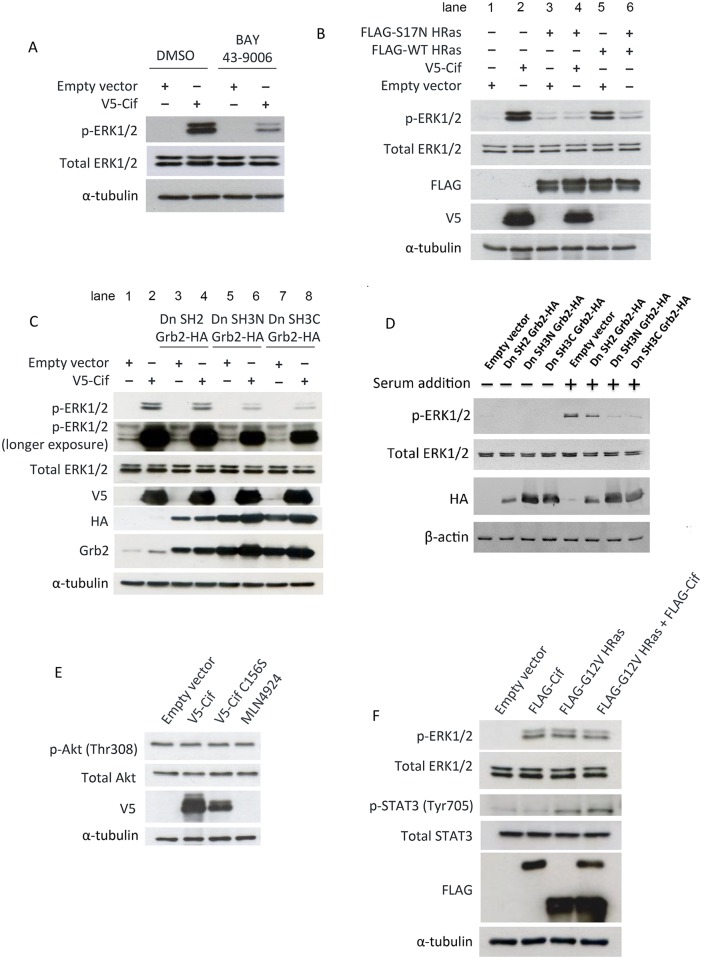
Cif likely activates MAPK/ERK signaling at the level of the Grb2-SOS1 complex. (A-C, E-F) Cells were transfected with the indicated expression plasmids for two days, and then treated with (A) 10 μM BAY 43–9006 or 0.1% DMSO for the last 6 hours or (E) 1 μM MLN4924 for the last 24 hours, followed by Western blotting of the cell lysates using the indicated antibodies. (D) Cells were transfected with the indicated expression plasmids for two days. The cells were then incubated for 6.5 hours in serum free medium, followed by re-addition of serum for 30 min, where indicated. The cell lysates were then analysed by Western blotting using the indicated antibodies.

We next determined whether Cif directly activates MEK or another kinase or signaling intermediate that is further upstream of MEK. We treated cells with the Raf inhibitor BAY 43–9006, which inhibits the kinase activity of C-Raf and B-Raf and thus blocks MEK and ERK phosphorylation. As shown in Fig 4A, inhibition of Raf activity with BAY 43–9006 significantly inhibited activation of ERK by Cif, suggesting that it is unlikely that Cif directly activates MEK. This result indicates that Cif may mediate its effect on ERK activation via Raf or a target that is upstream of Raf.

Therefore, to determine if Cif directly activates Raf, we used a dominant negative Ras mutant, S17N HRas, as an approach to inhibit Ras activity. It is generally accepted that S17N HRas exerts its effects by sequestering upstream activators and simultaneously being impaired in activating downstream effectors [20]. As a result, S17N HRas inhibits the activation of endogenous Ras and prevents downstream Ras signaling events. When we expressed FLAG-tagged S17N HRas, we observed that the dominant negative Ras completely blocked the effect of Cif on ERK activity (Fig 4B—compare lane 4 to 2). This result suggests that Cif functions at the level of Ras or upstream of Ras to mediate its effect on ERK activation.

The dominant negative Ras functions by sequestering the upstream Grb2-SOS1 complex. This suggests that ERK activation by Cif is dependent on a functional Grb2-SOS1 complex. In response to RTK activation, the Grb2 protein normally recruits SOS1 to the plasma membrane. Upon plasma membrane translocation, SOS1 can activate plasma membrane-bound Ras by functioning as a guanine nucleotide exchange factor (GEF) for Ras. Therefore, we next tested whether Cif requires plasma membrane translocation of the Grb2-SOS1 complex to activate ERK. To test this, we used dominant negative mutants of Grb2 that prevent the recruitment of SOS to the plasma membrane [21,22]. The Grb2 protein contains two Src Homology 3 (SH3) domains, one at the N-terminus and the other at the C-terminus of Grb2. The SH3 domains mediate the binding to a proline-rich region in SOS. The Grb2 protein also contains one Src Homology 2 (SH2) domain that is responsible for binding to specific phosphorylated tyrosine residues on activated RTKs. In our experimental approach, we used dominant negative SH2 and SH3 Grb2, in which the SH2 domain and the SH3 domains in Grb2 are inactivated by point mutations, respectively. Dn SH2 Grb2 is expected to bind to endogenous SOS1, but is unable to recruit SOS1 to RTKs. Hence, dn SH2 Grb2 sequesters SOS1 in the cytoplasm. In contrast, dn SH3 Grb2 is expected to bind to RTKs but not to SOS1. Consequently, dn SH3 Grb2 prevents binding of the endogenous Grb2-SOS1 complex to RTKs. As shown in Fig 4C, when we co-expressed dn SH3N Grb2 (N-terminal) or dn SH3C Grb2 (C-terminal) with Cif, Cif-induced ERK activation was markedly inhibited, as indicated by the pronounced decrease in ERK phosphorylation (compare lane 6 or 8 to lane 2). This result suggests that the effect of Cif on the MAPK/ERK pathway is dependent on the recruitment of SOS1 to the RTK. The dn SH2 Grb2 also inhibits the translocation of SOS1 to the plasma membrane. However, when we expressed dn SH2 Grb2, we only observed a slight inhibition of ERK activation by Cif (compare lane 4 to 2 in Fig 4C). It was noted, however, that compared to the dn SH3 Grb2 mutants, the expression level of dn SH2 Grb2 was lower. We thus tried to validate the dn Grb2 proteins by measuring their effects on growth factor-induced ERK1/2 activation. We found that dn SH3N Grb2 and dn SH3C Grb2 had a strong inhibitory effect, whereas dn SH2 Grb2 inhibited serum-induced ERK1/2 activation only weakly (Fig 4D). Hence, the effect of the various dn Grb2 constructs on growth factor-induced ERK1/2 activation correlates well with their effects on Cif-induced ERK1/2 activation. Thus, taken together, our results suggest that the activation of ERK by Cif is dependent on the recruitment of the Grb2-SOS1 complex to the plasma membrane via binding to RTKs.

Our results in Fig 4C raise the possibility that Cif functions by mediating RTK activation. To test this possibility, we examined the effect of Cif on the PI3K/Akt pathway, which is also downstream of RTKs. We found that Cif had no effect on the PI3K/Akt pathway as indicated by a lack of an increase in Akt phosphorylation at threonine 308 (Fig 4E). It is also known that growth factor signaling via RTKs can lead to the activation of the STAT3 pathway. However, we found that Cif transfection did not lead to STAT3 activation, as indicated by the lack of an increase in STAT3 phosphorylation at tyrosine 705, compared to the known activator of the STAT3 pathway constitutively active Ras (G12V HRas) (Fig 4F). These results indicate that Cif does not directly activate RTKs and suggest that Cif targets the Grb2-SOS1 complex downstream of RTKs to activate the MAPK/ERK pathway.

### Cif expression modifies SOS1 in a region containing the CDC25-H and proline-rich domains

**Fig 5:**
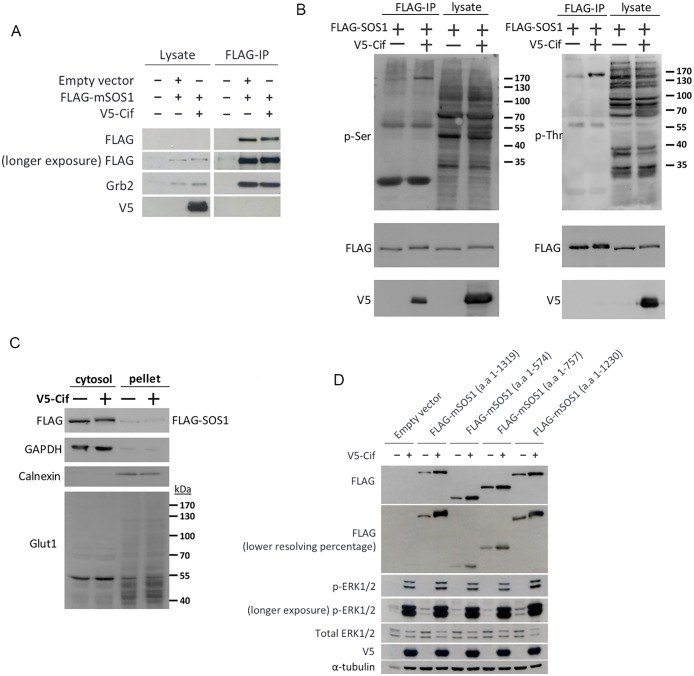
Cif expression regulates the CDC25-H and proline-rich domains in SOS1. (A,B) HEK293T cells were transfected with the indicated expression plasmids for two days, followed by FLAG immunoprecipitation of the cell lysates using FLAG-agarose and Western blotting of the immunoprecipitates and aliquots of the cell lysates using the indicated antibodies. (C) All cells were transfected with FLAG-SOS1 as well as V5-Cif or empty vector, as indicated, for two days. Cells were then lysed in hypotonic lysis buffer (25 mM Tris-HCL, 2 mM EDTA, 2 mM EGTA, 0.1% β-Mercaptoethanol, Roche protease inhibitor cocktail, pH 7.5) and cell lysates subjected to freeze-thawing at -80°C. The cytosolic fraction was separated from the remaining cellular compartments (membranes and nuclear fraction) through centrifugation. The proteins in the cytosolic supernatant and the pellet were denatured using SDS loading buffer and analysed using Western blotting with the indicated antibodies. Equal corresponding amounts of cytosol and pellet were loaded. GAPDH served as a control for the cytosolic fraction and calnexin as a control for the membrane fraction. Glut1 (55 kda) served as an additional control for the membrane fraction, as the higher molecular weight, glycosylated Glut1 species can only be detected in the membrane fraction. (D) Cells were transfected with the indicated FLAG-mSOS1 truncation constructs for two days, followed by Western blotting using the indicated antibodies. The bottom FLAG blot in the Western blot panel was obtained from running a duplicate set of lysates through another SDS-PAGE gel of a lower resolving gel percentage for a longer time to increase the resolving power.

Our results suggest that Cif exerts its effects on the MAPK/ERK pathway downstream of the RTKs, and that it potentially acts at the Grb2-SOS1 complex. Hence, we sought to characterize the potential mechanism through which Cif modifies the Grb2-SOS1 complex to activate ERK MAPK. Grb2-dependent recruitment of SOS1 to RTKs is required to activate the GEF activity in SOS1 to catalyze GDP to GTP exchange in Ras [23]. We hence hypothesized that Cif increases Grb2-SOS1 binding to promote SOS1 activity. Therefore, we examined the binding of Grb2 to SOS1 in the presence or absence of Cif by using coimmunopreciptation. As shown in Fig 5A, we were able to readily detect an interaction between transfected FLAG-SOS1 and endogenous Grb2. However, we found that there was no noticeable difference in the amount of Grb2 that was coimmunoprecipitated with SOS1 when Cif was present. This suggests that Cif does not alter Grb2-SOS1 binding to promote SOS1 activity. However, interestingly, we noted that the co-expression of SOS1 and Cif resulted in a slight band shift in SOS1 as seen in both the lysate and the FLAG IP lanes (Fig 5A), suggesting that Cif may possibly induce a posttranslational modification of SOS1 such as phosphorylation. Using phospho-serine and phospho-threonine specific antibodies, we indeed observed that Cif expression resulted in an increase in SOS1 phosphorylation at serine and threonine residues (Fig 5B). It was also noted that Cif mediated modification of SOS1 does not require membrane translocation of SOS1, but can also be observed with cytoplasmic SOS1 (Fig 5C). Furthermore, Cif expression did not lead to increased membrane translocation of SOS1 (Fig 5C), suggesting that Cif mediated modification of SOS1 may regulate the activity of the SOS1 proteins towards downstream effectors such as Ras.

The Ras guanine nucleotide exchange factor SOS1 has a multi-domain structure. The domain organization of SOS1 comprises of a histone fold domain, a Dbl homology (DH)-Pleckstrin homology (PH) domain, a Ras exchanger motif (REM), a CDC25-homology (CDC25-H) domain with GEF activity, and a proline-rich C-terminal region that binds Grb2 and links SOS1 to activated RTKs. The catalytic REM and CDC25-H domains govern the GEF activity of SOS1 on its target Ras protein. The histone fold domain and the DH-PH domain have been suggested to exert an autoinhibitory effect on the catalytic REM and CDC25-H domains [24,25]. It is also known that the C-terminal proline-rich region of SOS1 contains multiple phosphorylation sites that possibly play a role in the regulation of Ras activation by SOS1 [26]. Given the key role of these domains in the regulation of SOS1 function, we therefore hypothesized that Cif modulates the phosphorylation status of one of these domains to contribute to increased ERK activation. To investigate and map the phosphorylation sites, we generated a number of mouse SOS1 (mSOS1) C-terminal deletion constructs comprising amino acids 1–1230, 1–757, and 1–574. We then co-expressed each deletion mutant with Cif to examine which region of SOS1 is responsible for the band shift in the presence of Cif. We found that only the mSOS1 (a.a 1–1230) mutant retained the band shift pattern similar to full length mSOS1 with Cif co-expression (Fig 5D). In contrast, all other deletion constructs showed no band shift in the presence of Cif. This suggests that Cif likely modulates the phosphorylation status of mSOS1 in the region between amino acids 757 and 1230, which contains the CDC25-H and proline-rich domains.

## Discussion

Cif has previously been shown to inhibit CRL function [4]. CRL inhibition is due to a novel mechanism that involves a unique deamidation of Nedd8, a ubiquitin-like protein that is conjugated to cullin proteins and necessary for CRL activation. Here we provide evidence for a novel function of Cif that is independent of CRL inhibition but dependent on its deamidase activity. We found that Cif expression induces marked ERK phosphorylation and thus leads to the activation of MAPK/ERK signaling, indicating that Cif has an effect on cellular signaling. Importantly, our results suggest that Cif deamidase likely has other downstream targets in addition to Nedd8.

Our studies investigating the various MAPK/ERK pathway signaling intermediates and our finding of a potential Cif-induced SOS1 posttranslational modification suggest that Cif acts at the level of SOS1. Cif is known to have deamidase activity [4]. However, protein deamidation is not expected to cause a mobility shift in SDS-PAGE gels [4,12]. Furthermore, we were unable to detect a robust and reproducible interaction between Cif and SOS1 using reciprocal co-immunoprecipitation assays (data not shown). It should be noted, though, that enzyme-substrate interactions can be weak and transient. Nevertheless, our data suggest that Cif activates ERK *via* a novel mechanism that may involve SOS1 phosphorylation. Cif appears to induce the phosphorylation of a region in SOS1 that comprises the CDC25-H and proline-rich domains. The CDC25-H domain, together with the REM domain, mediates Ras nucleotide exchange. The C-terminal region has been suggested to exert a negative regulatory effect on the activity of human SOS1 [24,27,28]. Therefore, it is possible that by modulating the phosphorylation status of these two domains, Cif regulates SOS1 catalytic activity or alternatively downregulates or reverses the negative regulatory effects of the C-terminal region of SOS1. This would enhance the GEF activity of SOS1 and hence promote Ras activation and downstream signaling events. However, it should be noted that we currently cannot rule out that Cif functions to activate Ras via a different mechanism.

In future studies, it will be important to identify the phosphorylated amino acids in SOS1 and to characterize the mechanism through which Cif induces SOS1 phosphorylation. Cif-induced SOS1 phosphorylation may be due to deamidation of an upstream regulator of SOS1, such as a protein kinase. Alternatively, Cif may also deamidate a particular glutamine residue in SOS1, which in turn would prime the phosphorylation of SOS1 by an upstream protein kinase.

Finally, our results raise the interesting possibility of the existence of an upstream regulator of SOS1 that can lead to a marked activation of ERK signaling. Hence, another important question is to elucidate how SOS1 phosphorylation results in increased GEF activity. So far, SOS1 phosphorylation by different protein kinases has been found to mainly play a negative regulatory role on MAPK/ERK signaling [26,29]. Thus, identification of the mechanism underlying Cif-dependent ERK MAPK activation is likely to provide novel insights into the regulation of this important signaling pathway.

Whether Cif promotes the pathogenicity of *B*. *pseudomallei* is currently not well characterized, since not all *B*. *pseudomallei* isolates possess Cif. It was originally proposed that Cif promotes the intracellular bacterial replication by inhibiting the turnover of epithelial host cells [18]. This effect is due to cell cycle arrest as a result of the Cif-mediated inhibition of CRL-mediated degradation of cell cycle inhibitors such as p27. However, in a recent paper by McCormack *et al*. [30], it was demonstrated that during *Yersinia pseudotuberculosis* infection, Cif plays a role to inhibit the activity of perforin-2, a mediator of the innate immune response of host cells. The authors found that upon *Y*. *pseudotuberculosis* infection, perforin-2 becomes activated through monoubiquitination. Perforin-2 ubiquitination is mediated via the CRL SCF^**β**-TrCP^ and this results in the translocation of perforin-2 to cellular membranes, in particular the plasma and endosomal membranes. Upon translocation, perforin-2 then exerts its bactericidal activity by inducing lysis of plasma membrane-bound or endosome-encapsulated bacteria. Cif mediated CRL inhibition prevents perforin-2 monoubiquitination and activation [30]. Importantly, the authors demonstrated that *in vivo* Cif increases *Y*. *pseudotuberculosis* virulence in wild type but not perforin-2 deficient mice. This indicates that inhibiting the perforin-2 dependent host response is the primary function of Cif in *Y*. *pseudotuberculosis*.

However, the described mechanism in *Y*. *pseudotuberculosis* infection is unlikely to be of major relevance during the infection with Cif-expressing *B*. *pseudomallei*. This is because *B*. *pseudomallei* can readily escape from the endosomes into the host cytosol [31], thus avoiding the bactericidal activity of perforin-2. As such, it is likely that perforin-2 does not play a major role in the response of host cells to *B*. *pseudomallei* infection. Our study suggests an alternative mechanism through which Cif may potentially exert its cellular effect. Prolonged CRL inhibition is known to induce cellular apoptosis. This is partly due to prevention of the degradation of key cell cycle regulatory proteins, including the important DNA replication licensing factor CDT1. Failure to degrade CDT1 leads to DNA re-replication and induction of cellular apoptosis [32]. Given that host cell apoptosis would also limit bacterial replication and survival, we hypothesized that Cif may counteract the pro-apoptotic effects of CRL inhibition by activating cellular anti-apoptotic pathways through ERK activation. We indeed observed that Cif expression increased the phosphorylation of the most abundant Bim splice variant Bim_EL_ in an ERK1/2-dependent manner. ERK1/2-dependent phosphorylation is well known to induce proteasome-dependent Bim_EL_ protein degradation. Surprisingly, despite inducing marked Bim_EL_ phosphorylation, Cif reduced the Bim_EL_ steady state protein levels only slightly. It may be necessary to carry out half-life measurements of endogenous Bim_EL_ protein to detect Cif-induced changes in Bim_EL_ protein stability. We also observed that Cif increased the expression of the anti-apoptotic protein Mcl-1 and the phosphorylation of Bad, leading to inhibition of its pro-apoptotic function (see Fig 2C). However, these effects were largely ERK independent as they were not or only slightly inhibited in the presence of MEK inhibitor. It remains to be seen whether Cif is expressed to a sufficient level to trigger these changes during bacterial infection. If so, future studies to test whether Cif-dependent ERK MAPK activation and the induction of anti-apoptotic pathways is important to prevent host cell apoptosis and promote *B*. *pseudomallei* virulence would be of interest.

In conclusion, we have identified a novel deamidation-dependent mechanism of action of the *B*. *pseudomallei* virulence factor Cif to activate MAPK/ERK signaling. The elucidation of the exact molecular mechanism is expected to give important new insights into the regulation of the MAPK/ERK signaling pathway. Our study demonstrates that bacterial proteins such as Cif can serve as useful molecular tools to uncover novel aspects of mammalian cellular signaling pathways.
